# Climate change and its impact on children and adolescents sleep

**DOI:** 10.1016/j.jped.2024.10.009

**Published:** 2024-12-12

**Authors:** Maria Cecilia Lopes

**Affiliations:** a Universidade de São Paulo, Faculdade de Medicina, Instituto da Criança, Unidade de Polissonografia, São Paulo, Brazil; b Universidade de São Paulo, Instituto e Departamento de Psiquiatria, Programa de Transtornos Afetivos da Infância e Adolescência (PRATA), São Paulo, Brazil

**Keywords:** Climate change, Sleep, Greenhouse effect, Pediatrics

## Abstract

**Objective:**

This review discusses the impact of climate change on sleep, anxiety, and eating in the pediatric population.

**Data source:**

This is a nonsystematic literature review based on a search using PubMed and MeSH terms in titles and abstracts with these keywords: climate change, sleep, greenhouse effect, children, and adolescents.

**Data synthesis:**

Climate change events are associated with human intervention in the ecosystem, having a strong impact on cognitive functions, physical and mental health, as well as subjective well-being, particularly in youth. Climate change is caused by human activity with changes in the composition of the global atmosphere caused by emissions of gases, such as carbon dioxide, which increase the greenhouse effect. This review discusses the impact of climate change on sleep, anxiety, and feeding in the pediatric population.

**Conclusions:**

Early detection of vulnerability conditions, along with adaptation strategies is necessary to address climate stressors with a focus on healthy sleep and eco-anxiety. Pediatrics has an important role to play in protecting healthy sleep in children.

## Climate change and society

The climate change observed on all continents is caused primarily by greenhouse gas emissions from natural systems and human activities.^1^ Household energy consumption accounts for around 72% of global greenhouse gas emissions (with the remainder coming from public, nongovernmental, and business sources).^2^^,^^3^ Recurring events can be anticipated, characterizing the adaptive capacity of each species, a phenomenon known as “evolution”, which modulates the internal temporal systems. Climatic events are associated with the way society deals with temporal organization, with an impact on cognitive functions, physical and mental health, as well as subjective well-being.^3^ Climate change is closely associated with human activity with changes in the composition of the global atmosphere, caused by emissions of gases, such as carbon dioxide, which increase the greenhouse effect. This phenomenon was described by Joseph Fourier in 1824,^4^ with the “greenhouse” effect initially described as essential for survival on the planet. In the 1820s, still at the beginning of the Industrial Revolution, Fourier made a major contribution to addressing this issue. Fourier identified a balance between the amount of energy from the Sun absorbed by Earth and the amount of energy that the Earth re-emits to the universe. According to this balance, the temperature of the Earth should be much lower than it is. Fourier then speculated that the atmosphere retains heat to maintain its temperature, functioning like a blanket or greenhouse. Fourier predicted the greenhouse effect, although he did not give it this name. It is known that the intensity of the greenhouse effect is directly related to the chemical composition of the atmosphere.^5^ Apparently, the current composition of the atmosphere is a product of the long evolutionary history of life on Earth, and microorganisms probably determined the basic composition of the atmosphere since the origin of life. Thus, the symbiosis is such that the chemical composition of the atmosphere promotes the conditions for life, and this regulates the chemical composition of the atmosphere.^6^, ^7^, ^8^

The Industrial Revolution resulted in the industrial scale of increased carbon dioxide production with increased energy production. In 1896, Chemist Svante Arrhenius described the relationship between the increase in carbon dioxide and the increase in the greenhouse effect.^9^ It is now well understood that we are generating climate instability that causes climate catastrophes, resulting in more frequent and intense extreme disasters related to natural climate change, such as forest fires, storms, and floods resulting from extreme heat with increased temperature and droughts, and climate and environmental changes leading to dry weather. Long-lasting climate change is observed in landscapes and physical environments caused by rising sea levels and altered ecosystems induced by humans, and is, therefore, attributed directly or indirectly to human activity that alters the composition of the global atmosphere, in addition to the natural climate variability observed in periods of seasonality.

The climate has been changing in recent decades, affecting the health and well-being of children around the world. Events related to climate change affect the health and well-being of children and adolescents, including children's mental and physical health, nutrition, safety, and protection, learning opportunities, and family care and connection.^10^ Sleep and climate are related to the variable of time. From this perspective, the chronological time of sleep is determined by circadian factors that correspond to the Earth's rotation cycle. The climate depends on seasonal factors that interact with environmental conditions according to the time range studied. Climate can also be described as a long-term pattern of weather conditions in a specific location. Climatic elements and factors include solar radiation, temperature, humidity, pressure, winds, precipitation, topography, and sea currents. The globe is warmer with intense meteorological changes due to multiple integrated factors. Climate can also be defined as a set of weather types that generate an average to define the climate of the region, whose changes in meteorological systems result from sea currents and wind currents with extreme events that change the definition of weather and climate, modifying other factors such as temperature, pressure, air mass, rainfall patterns, latitude, altitude, vegetation, and relief. Climate events mainly affect individuals with social and physical vulnerability, and gender differences, mirroring inequality and social disparities. Pediatrics plays a critical role in raising awareness of new behaviors and more sustainable models.

## Weather events and pediatric sleep

Changes in the climate lead to changes in sleep perception, with acute and chronic consequences for sleep in all age groups. Sleep is a reversible behavioral state of environmental perception with apparent nonresponsiveness followed by wakefulness, characterizing arousability.^11^ From the period of sleep onset until awakening, sleep instability is observed, which is the result of sleep maintenance mechanisms that act contrary to the forces that promote awakening.^12^ Sleep is necessary for restoring wakefulness processes, influencing cognitive activity and emotions, and acting on physical and mental well-being in all age groups.^11^

Climate change generates events that promote changes in sleep perception, as well as increasing sleep disorders,^10^ modifying the sleep of the pediatric population. Sleep in pediatrics is considered vital for development,^13^ and is essential for protecting cognitive activities and restoring synaptic activities. It is well established that sleep is of fundamental importance for health and well-being, including memory consolidation,^14^ regulating the immune system^15^ and restoring energy levels.^16^ Adverse health outcomes, such as diabetes and cancer, have been associated with sleep disorders and poor sleep quality.^17^, ^18^, ^19^

Sleep is a period of rest of the body associated with brain activities that change according to age group. There are often several individuals of different age groups in a given family environment, each with their particularities, defining a setting referred to as the ecology of sleep, in which children, parents, siblings, and grandparents cohabit in the same environment.^12^ Sleep must be studied and observed in all age groups, and this increases the challenges for families experiencing events caused by climate change. The association of sleep duration with health is complex, since both short (generally defined as ≤ 5 or < 6 h) and long (generally defined as ≥ 9 h) sleep duration have been associated with adverse health outcomes.^17^ While measures of sleep duration are relatively straightforward, the concept of sleep quality is more complex, as is the concept of sleep instability. It is recognized that climate events modify sleep quality, and this topic will be covered in this review of research on sleep adaptation in relation to the new climate paradigms we will face in the coming years.^10^^,^^20^

## Ontogenetic changes in sleep

**Figure 1:**
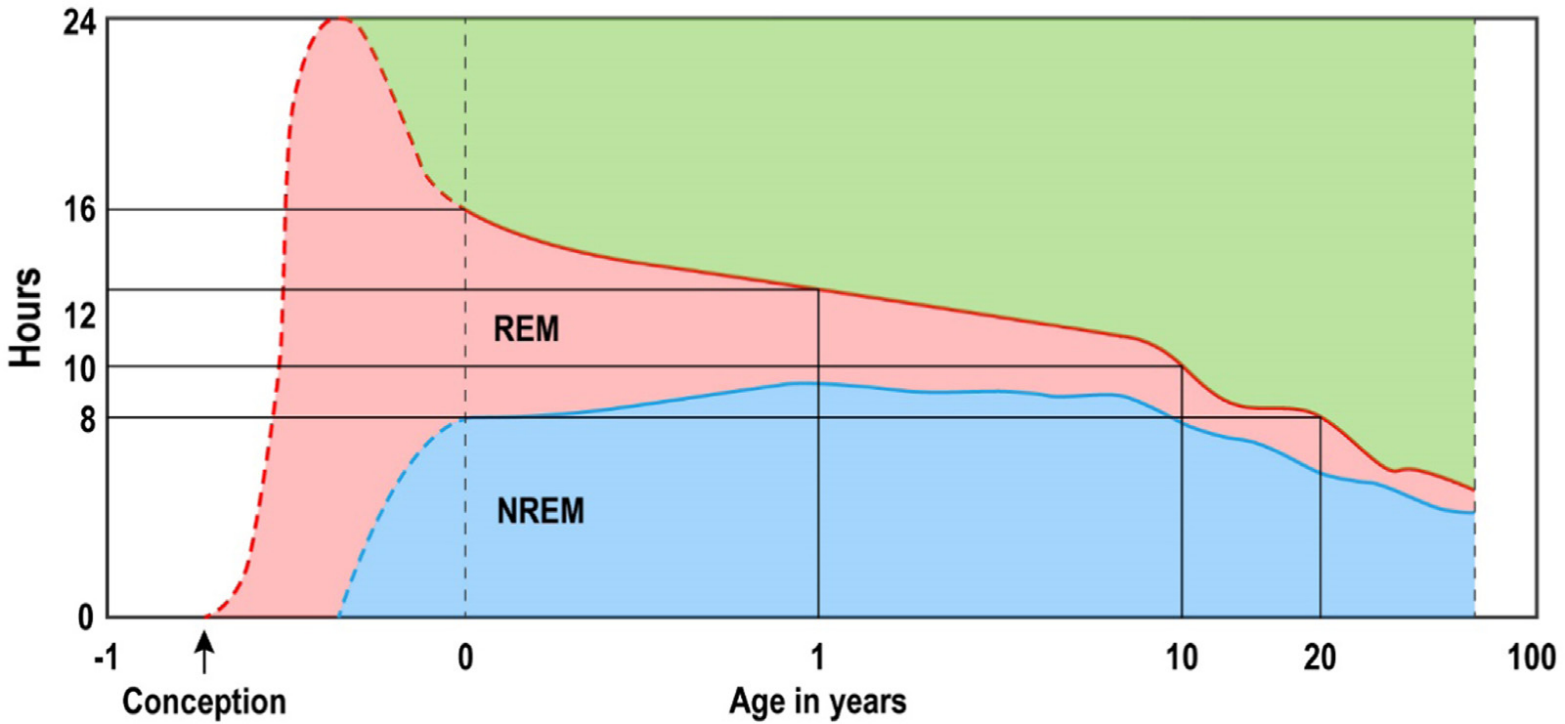
Expression of delta sleep and REM sleep according to pubertal development.^12^^,^^22^

Sleep changes throughout childhood growth and development. Sleep in the neonatal period of up to 6 months can be characterized as a manifestation of the development of brain rhythms. From 6 months onwards, wave patterns continue to change according to brain maturation, which is closely linked to the development of delta sleep, which is associated with synaptic expression. The peak of delta sleep development is around 10 years of age,^21^ when the pubertal growth spurt occurs, as well as when a decrease in neurons and a consequent decrease in synaptic interactions occur. REM sleep, in turn, does not present quantitative changes in adolescence. The expression of delta sleep and REM sleep, according to the development of puberty, is shown in Figure 1.^22^ Subjective and objective sleep analysis contribute to a better understanding of healthy sleep and will be important tools for promoting the health of children and adolescents affected by climate change.

## Sleep in pediatrics: The protective pathway for neurodevelopment

According to Nathaniel Kleitman,^21^ our basal state is, in part, a product of sleep, and we wake up to feed ourselves, procreate, and maintain our brain activity in contact with the external environment and our internal environment, or rather with our endogenous factors. The first hour of life is marked by the search for breastfeeding, with feeding being our first pacemaker of extrauterine life in skin-to-skin contact.^23^ We seek food regardless of the maturity of the visual system. Like birds that sing as an immediate survival reflex, the food search is a reflex and survival instinct supporting the development process. The chronobiological sleep pacemaker in newborns is still immature, with polyphasic sleep being observed, with several cycles in 24 h, generally starting with REM sleep (rapid eye movement), which is closely linked to the limbic system, modulated by the ascending reticular system located in the brainstem and pontine nuclei also located in the brainstem.^22^ Babies go through different phases of sleep with active sleep (REM sleep) and calm sleep (Non- REM sleep; NREM sleep) that correspond to deep sleep, composed of slow waves, with a restorative component associated with synaptic plasticity.^24^ During the first months of life, this modulation may be associated with motor development, which demonstrated a relationship between sleep and motor delay only in the first year of life in extremely premature infants,^25^ indicating a target for intervention to protect neurodevelopment. In the first months of life, REM sleep decreases, and slow-wave sleep increases, peaking at 10 years of age, preparing for synaptic pruning during puberty, which is also associated with hemispheric specialization of decision-making, sensations, and emotions. Children are morning people; that is, they go to bed early and tend to wake up early. When we have a child who is not sleeping early, there are environmental factors that must be modified to promote healthy sleep.

## Sleep and its intimate relationship with the climate

The data on temperature is clear: the authors have seen a continuous increase in temperature above the preindustrial average of 1850–1900; for example, there has been an increase of 1.7 °C since 1948 in Canada.^2^ “Heat waves, extreme heat and climate change”^8^ are associated with extreme weather conditions. Reducing emissions has become necessary, as has removing carbon dioxide, and restoring forests in biomes around the world. Global temperature will increase by 1.5 °C by the end of this century, and without proper control, they could increase by 3 °C or 4 °C.^9^ Sleep duration will decrease as temperatures rise. Insufficient sleep, in turn, alters cognitive performance, reduces productivity, compromises immune function, harms cardiovascular health, increases depression, anger, and suicidal behavior.^26^ Therefore, understanding the physiology of sleep will be essential for pediatric interventions in response to sleep changes caused by global warming.

The origin of sleep is interrelated with the origin of life. The resting state that follows the active state is present in several species and is an important mechanism for repairing and restoring cellular metabolism. The glymphatic system filters impurities that can cause harmful inflammatory processes in the brain. Sleep is a wonderful journey, and what makes it even more extraordinary is a simple fact: we never know that we are actually sleeping or when we are sleeping. It is impossible to have conscious and experimental knowledge of the dreamless sleep phase. Furthermore, we have great difficulty in monitoring the exact moment in which we are asleep without the help of neurophysiological procedures. At certain moments during sleep, the brain seems to be more active than during wakefulness, consuming large amounts of glucose and oxygen, while neurons fire rapidly. While we sleep, our mind assumes a different consciousness and lives in a world that is as complex as the world we live in when we are awake.

Sufficient sleep is essential for effective memorization, decision-making, and academic and athletic performance. Learning is a cognitive activity resulting from memory consolidation, and sleep is of fundamental importance in this process of formatting memories. This memorization process is affected by adaptive mechanisms that seem to be determined by circadian phenotypes that are natural genetically established tendencies that promote sleep better and avoid physiological harms of sleep deprivation. This memorization process during sleep is affected by individual phenotypic differences and a range of environmental factors such as eating habits and engaging in complex learning tasks before bedtime. Cyclical events occur during sleep, with NREM sleep and REM sleep being observed, the latter with activity similar to wakefulness, but with muscle atony. REM sleep is associated with dreams that influence memory processes, and processes in NREM sleep associated with synaptic neuroplasticity are recognized. The presence of synaptic activation may be responsible for allowing processes to be recorded that make memory consolidation during sleep viable.^12^

With rising temperatures, changes are expected in proteins that regulate cellular energy balance and alterations in temperature-sensitive signal transduction cascades,^27^ and temperature-sensitive ion may change due to physiological responses to thermal extremes.^8^ Living organisms may exhibit plasticity in response to heat, repressing gene expression and increasing membrane fluidity.^28^^,^^29^ In other words, extensive physiological adaptation will be required to maintain membrane stability in response to increasingly frequent heat waves. Undoubtedly climate change will modulate behavior, with changes also in mood due to reduced sleep time. Mood disorders are common and affect more than 120 million people around the world, with physical, mental, social, and economic impairment. The impact of mood disorders on society includes suicidal tendencies, a process that may be modulated by sleep.^30^ Also, recognizing conditions of social vulnerability early on becomes essential for addressing mental health in childhood and adolescence, in which sleep is essential, with easy detection of sleep disorders and a broad and effective therapeutic approach. In climate events, sleep is one of the factors most affected. Pediatrics needs to be prepared to carry out immediate interventions with the application of sleep hygiene measures (Table 1)^31^ and strategies to facilitate more stable sleep and the longest possible duration.

**Table 1:** Habits to improve sleep health.

• Keep relatively consistent bedtimes and wake-up times. Changes in sleep habits, such as going to bed later on weekends, can disrupt sleep. • Sleep only as much as necessary. Staying awake and lying in bed for long periods of time does not improve the quality of your sleep. • The bedroom should not be used for working, studying, or eating. • People with insomnia should avoid reading (particularly on a computer or phone screen) and watching television immediately before going to bed. • Do not nap during the day without a medical prescription. • Physical exercise should be done at most 4 to 6 h before going to bed. • Relax your body and mind 60 to 90 min before going to bed. Never try to solve problems before going to sleep. • Do not drink coffee, black tea, chocolate, or any stimulating drinks after 5pm. • Although alcoholic beverages help you relax, they can disrupt the quality of your sleep. People who snore should avoid them, as they can worsen snoring and breathing pauses, as a result of the relaxation caused by alcohol in the respiratory muscles. • Do not smoke before going to bed, as nicotine causes insomnia and nonrestorative sleep. • Avoid eating just before sleep, eat a lighter meal at dinner time. Balance this by eating a heavier meal at breakfast or lunch. • Excessive heat and cold significantly affect sleep, so keep the bedroom at a pleasant temperature. Bedroom temperature is best at 66.2°F (19 °C); if not, 68–77°F (20–25 °C); indoor temperature >77°F (25 °C) not recommended for sleep.^31^ • Noise can cause poor sleep. Modify your bedroom to prevent unnecessary noise.

## Climate, sleep, anxiety and mood disorders

**Figure 2:**
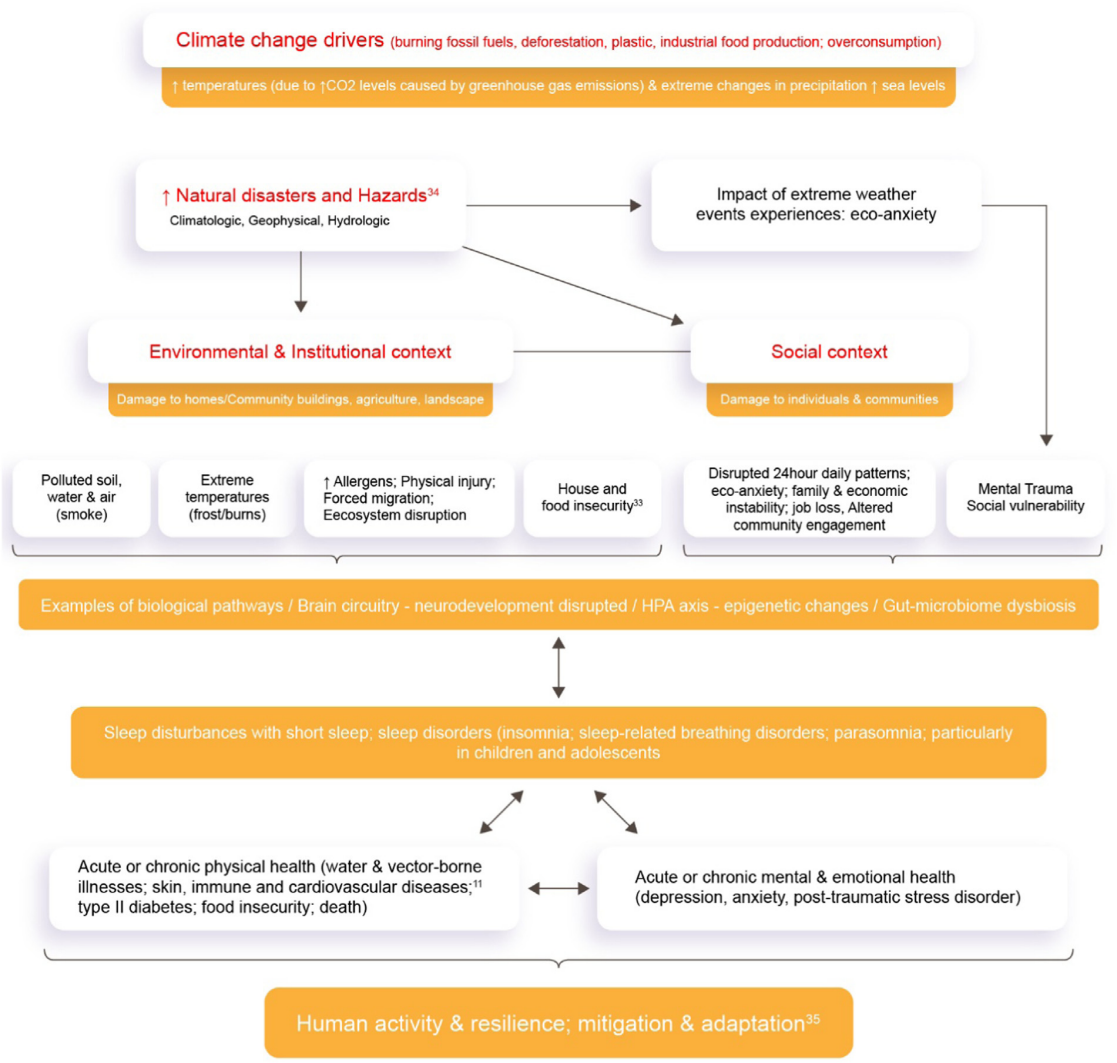
Relationship between sleep, anxiety, and climate change according to Gaston et al.^34^ and Helldén et al.^35^

Anxiety about climate change can be described as a vague and unpleasant feeling of fear, apprehension, characterized by tension or discomfort derived from anticipation of danger, something unknown or strange about climate-related issues.^32^ However, unlike adults, children may not recognize their fears as exaggerated or irrational, and it has been reported that children and adolescents may experience eco-anxiety and negative affective responses in response to awareness of climate change, including depression, anxiety and extreme emotions such as sadness, anger and fear.^33^ Clearly, there is a relationship between climate change, anxiety and decreased sleep time (Figure 2).^34^^,^^35^

The projection of insufficient sleep, when associated with changes in nighttime temperature, and the impact of environmental warming on insufficient sleep is observed cumulatively and in short sleep attributed to temperature for all countries. Consistent with the literature on climate impacts, and increasing concentrations of atmospheric greenhouse gases and linked to temperature projections, there is an annual excessive loss of individual sleep due to nighttime temperatures, with temperatures reducing an estimated average of 44 h of sleep per person annually.^26^ There may be a strong relationship with heredity, illustrated by several people in the same family experiencing anxiety about climate change and its impacts. In experiments on adults in which environmental temperatures were reduced, there was an annual increase of 11 additional nights of sleep.^26^ Total annual sleep loss due to warming nighttime temperatures may increase steadily until mid-century, with annual losses becoming significantly greater by 2099 in a scenario of increasing greenhouse gases. This phenomenon, identified as “sleep erosion” due to climate issues associated with rising temperatures, will have increasing impact on sleep due to the number of short nights of sleep attributed to rising temperatures. Such prospects increase anxiety about climate events and highlight the need for further studies on sleep protection and sleep maintenance.^26^

## Sleep deprivation due to the effects of climate change

**Figure 3:**
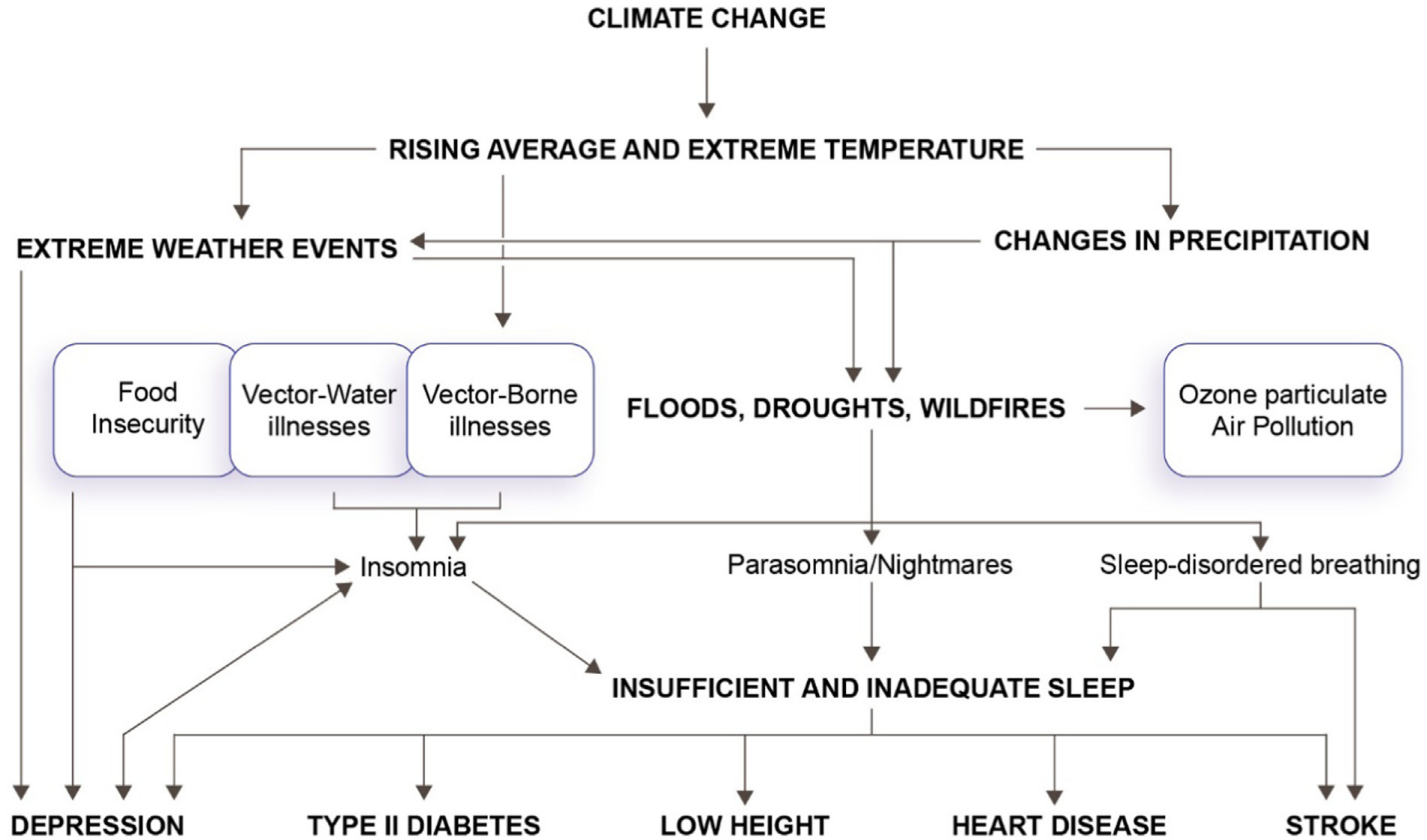
Relationship between climate change and sleep disorders according to Rifkin et al.^10^

Children are vulnerable to the health impact of disasters associated with climate events, with greater sensitivity to pollution and increased risk of physical and sexual abuse in shelters resulting from migration (Figure 3).^10^ This vulnerability is associated with free play, exposure to pollutants leading to bronchial hyperreactivity, developmental changes that impair weight and height growth, higher energy requirements from food than in adults due to a need for greater food intake per unit of body weight, sensitivity to trauma, which can affect sleep in childhood,^33^ and an increase in risk factors for physical and mental health disorders that begin in childhood and worsen in adulthood.^36^^,^^37^ Therefore, routine pediatric assessment of trauma related to climate change as an adverse or traumatic experience in childhood is necessary, as well as increased attention to the impact of climate change on sleep health throughout life.^33^

## Climate change, eating healthy, and sleep patterns

Healthy eating and sleeping are determining factors in the quality of life of the pediatric patients. Climatic events reduce sleep duration, increase sleep disorders, and lead to reduced food supplies necessary for full growth and development in childhood and adolescence. Understanding the close relationship between eating and sleeping can be a support strategy in climatic events that affect food distribution.

Sleep patterns improve after consuming tryptophan, a precursor of serotonin and an amino acid present in foods such as milk. Likewise, tryptophan depletion has been shown to reduce sleep quality. The mechanism for this revolves around tryptophan competing with other major neutral amino acids (e.g., valine, leucine, isoleucine, tyrosine, and phenylalanine) to cross the blood-brain barrier, where it is converted to serotonin, the precursor of the “nighttime hormone,” melatonin, released by reduced ambient light. We may ask ourselves: If a baby is not properly fed, will he or she have any difficulty sleeping? The question can be understood from the opposite perspective: If an infant is fed excessively at night, there is a risk of bronchoaspiration, gastroesophageal reflux, and increased nocturnal awakenings. Milk protein allergy is one of the causes of insomnia in infants. Reducing the nocturnal eating period is a well-tolerated dietary approach to caloric restriction, and it would be interesting to evaluate its long-term effects on sleep in children and adolescents. These effects suggest that nutrition affects health not only through the quantity or quality of intake, but also through the timing of food consumption according to the circadian cycle.

More broadly, it would be interesting to monitor and compare other health behaviors and markers, such as sleep quality or physical activity with eating schedules, according to the individual circadian cycle. In fact, smartphones can help with monitoring to provide detailed information on lifestyle behaviors according to the circadian clock. Consistent eating routines and sleep routines are essential. Sleep hygiene, eating following well-established routines, and physical activity are excellent strategies for maintaining healthy sleep (Table 1). Homeostatic factors, circadian factors, and conditions that generate hyperexcitability are associated with screen time and activities performed at night. Psychoeducation about habits and lifestyle encourage healthy sleep patterns for the entire family. Foods with antioxidants are recommended, as well as probiotics and prebiotics. Excessive consumption of sugar and ultraprocessed foods should be avoided as they cause inflammatory processes in the intestine, affecting mental health through the brain-intestinal axis. Certain foods promote neuromodulation through the expression of cholecystokinin (one of the first gastrointestinal peptides, acting as a neuromodulator and signaling center of the brain-gut axis, mediating emotion, digestion, and memory regulation). Improving sleep and nutrition leads to full hippocampal functioning with a positive effect on memory and expression of emotions.^38^ Considering repercussions in pediatrics due to climatic events, it is imperative to evaluate dietary habits associated with sleep hygiene.

## Final considerations

The main objective of this article is to identify the direct relationship between climate change and children's sleep. Climate events alter sleep, mental health, and eating habits in pediatric patients. The decision to reduce carbon emissions resulting from household supplies is correlated with the consumption behavior of families, expressed according to financial or physical conditions. Household consumption includes all areas of personal consumption in housing, mobility, food and other consumption (such as clothing, furniture, electronics), which depend on decision-making regarding behavioral changes that can affect sleep in children,^29^ as well as the need to protect sleep, with care regarding stimuli before bedtime and care regarding the sleeping environment.

Regulation is necessary to mitigate climate change and invest in prevention to avoid extreme weather events. Mitigating climate change will need to be the primary focus, but we will also have a migration crisis and the need to prepare coastal cities for the impacts on the economy, mental health and sleep resulting from extreme weather events. Individual and collective efforts are essential, together with government and organizational commitments to decouple economic well-being from increased emissions, with information about climate mitigation and adaptation, encouraging and supporting families to become active agents of decarbonization.^39^

Monitoring the effects of climate change should be universal, especially in pediatric populations, with consideration of diversity of socioeconomic status, racial/ethnic issues, and gender differences, with a focus on the role of sleep in climate change-related events on physical and mental health across the lifespan. These efforts will increase the capacity to incorporate healthy sleep into climate change adaptation, mitigation, and resilience strategies. Addressing pediatric sleep must be included in postdisaster programs, with appropriate indications for treatment, recovery, and resource allocation. Early detection of vulnerability conditions can inform adaptation strategies such as air conditioning, energy security programs, maintenance of good hydration, and psychoeducational programming. Training healthcare workers to address climate stressors with a focus on healthy sleep and addressing eco-anxiety is critical. The world needs help, and pediatrics has an important role to play in protecting healthy sleep in children.

## Funding source

This research did not receive any specific grant from funding agencies in the public, commercial, or not-for-profit sectors.

## Conflicts of interest

The author declares no conflicts of interest.
